# The Effect of the Hepatitis B Virus Surface Protein Truncated sC69^∗^ Mutation on Viral Infectivity and the Host Innate Immune Response

**DOI:** 10.3389/fmicb.2019.01341

**Published:** 2019-06-12

**Authors:** Kuanhui Xiang, Yiwei Xiao, Yao Li, Lingyuan He, Luwei Wang, Hui Zhuang, Tong Li

**Affiliations:** Department of Microbiology and Infectious Disease Center, School of Basic Medical Sciences, Peking University Health Science Center, Beijing, China

**Keywords:** HBV, truncated mutant, sC69^∗^, viral infectivity, innate immune response

## Abstract

Viruses could rapidly diversify into variants, which has long been known to facilitate viral adaption in the host. Recent studies showed that cooperation among variants and wild-type (WT) also increased viral fitness. Here, a mutant of sC69^∗^ in small hepatitis B surface protein (SHBs) that resulted in premature stop was investigated and the frequency of sC69^∗^ was 4.37% (19/435), most of which coexisted with the WT (78.95%, 15/19), indicating mixed viral populations. Functional studies showed that sC69^∗^ mutant was associated with lower viral spread, but could be rescued by coexisting with the WT. The sC69^∗^ mutant showed to attenuate host innate immune response during infection and poly (I:C) treatment such as IL29, ISG15, and RIG-I (*p* < 0.05). The lower immune response was not caused by the lower replication of sC69^∗^ mutant. Our data provide information that sC69^∗^ coexisting with the WT might facilitate the fitness and persistence of the viral quasispecies in the host.

## Background

Hepatitis B surface antigen (HBsAg) is a major viral protein of hepatitis B virus (HBV) circulating in patient serum and serves as an important virological marker for the evaluation of chronic HBV infection and antiviral response. We found that 4.37% (19/435) of the studied patients with genotype C HBV chronic infection carried the small hepatitis B surface (SHBs) sC69^∗^ mutant resulting in the truncated SHBs [about 1/3 length of wild-type (WT) SHBs]. This mutant usually existed in older people with lower HBV DNA levels. In addition, we found that the sC69^∗^ negatively impaired viral replication, infectivity and spread, which could be rescued by the WT. On the contrary, the sC69^∗^ mutant inhibited the host innate immune response more potently than that of the WT. These results indicate that HBV sC69^∗^ coexisting with WT might facilitate the viral fitness and surviving from the host surveillance.

## Introduction

Despite the availability of an effective vaccine and the improvement of antiviral treatment, chronic HBV infection remains a severe public health problem. According to the global hepatitis report in 2017 from World Health Organization (WHO), estimated 257 million people worldwide are chronically infected, with an increased risk for developing liver cirrhosis and hepatocellular carcinoma (Liang T.J. et al., 2015).

Hepatitis B virus surface protein consists of three related yet different proteins, the large, middle and small hepatitis B surface proteins (LHBs, MHBs, and SHBs, respectively). The SHBs has a MHR, including the ‘a’ determinant from AA 124–147, which is a dominant neutralizing epitope and most neutralizing antibodies target to (Locarnini et al., 2012). Naturally occurring AA variations in this region are prevalent and affect HBsAg antigenicity, secretion, circulating HBsAg and HBV DNA levels (Alavian et al., 2013). Huang et al. (2012) reported that the AA variations in the ‘a’ determinant could result in lower serum HBsAg levels in patients with occult HBV infection (Wu C.C. et al., 2012).

However, HBsAg AA variations naturally exist not only within the ‘a’ determinant region, but also within its flanking regions (Warner and Locarnini, 2008; Alavian et al., 2013). Recently, it was shown that HBsAg truncated mutants such as sW172^∗^, sW182^∗^, and sW199^∗^ occurred in patients both with and without antiviral treatment (Warner and Locarnini, 2008; Pollicino et al., 2012). Apart from these findings, another truncated mutant (sC69^∗^) was identified from CHB patients as shown in previous studies (Betz-Stablein et al., 2016; Xiang et al., 2017). Recently, the sC69^∗^ was found in genotype A CHB patients, which was reported to be associated with entecavir (ETV) and tenofovir (TDF) resistance (Shirvani-Dastgerdi et al., 2017). Interestingly, these truncated mutants could inhibit viral replication and secretion (Warner and Locarnini, 2008; Xiang et al., 2017). Our previous study showed that the sC69^∗^ mutant could restrict viral replication and secretion, but these defects could somehow be rescued by the WT HBs (Xiang et al., 2017). However, little is known about the influence of the sC69^∗^ mutant on viral infectivity and spread. Yan et al. (2012) reported that NTCP was a receptor for HBV infection, and NTCP overexpression in HepG2 cells was shown to confer susceptibility to HBV infection. Thus, it is possible to use HepG2-NTCP cells as an HBV infection model to study the influence of sC69^∗^ on viral infectivity and spread.

Recently, many studies have suggested that cooperative interactions among viral quasispecies (among variants and WT) support viral population fitness. For example, it was reported that a population with poliovirus variants was required for pathogenesis, suggesting that cooperative interactions among the variants and WT played important roles in viral fitness and survival (Pfeiffer and Kirkegaard, 2005). More recently, the cooperative interactions were also reported in measles virus and Coxsackie virus (Shirogane et al., 2012; Borderia et al., 2015). Similarly, it was also shown that HBV WT rescued the replication and secretion of variants such as sW172^∗^, sW182^∗^, and sC69^∗^ (Warner and Locarnini, 2008; Pollicino et al., 2012; Xiang et al., 2017). However, the reason why HBV replication-deficient variants exist in host or the significance of their coexistence with the WT is still not clearly understood. Our hypothesis for sC69^∗^ is that it can inhibit host innate immune responses, which does not only protect itself but also the WT from host immune surveillance.

As reported before, host immune response against HBV infection is crucial for better understanding of the pathological processes and viral elimination to control HBV infection (Rehermann and Nascimbeni, 2005). Lucifora et al. (2014) reported that APOBEC3A/3B cytidine deaminases induced by interferon (IFN) could deaminate cytosines in HBV cccDNA and lead to cccDNA degradation. Shlomai et al. (2014) reported that HBV infection could induce the IFN stimulated genes (ISGs) response in primary hepatocytes cultured in micro-patterned co-cultures of primary human hepatocytes with stromal cells. Yan et al. (2015a) showed that the IFN inducible protein, tetherin, inhibited HBV secretion. However, HBV has many strategies to escape from innate immune surveillance. For example, Liu S. et al. (2015) reported that HBsAg and hepatitis B e antigen (HBeAg) could inhibit major vault protein to negatively impair response to IFN therapy. In addition, HBV polymerase was shown to inhibit K63 ubiquitination of STING, thereby negatively impacting the innate immune response (Liu Y. et al., 2015). Interestingly, Chen et al. (2015) showed that HBV spliced variants were associated with negatively impaired response to IFN therapy. It was recently reported that cooperative interactions among two HBsAg mutants at different ratio could increase HBV replication and influence the host immune response and antibody response (Cao et al., 2014). We therefore wondered if the sC69^∗^ mutant could also inhibit innate immune responses and promote HBV persistence in the host.

To characterize how the sC69^∗^ mutant exists in the host, we sequenced the SHBs coding region in HBV genotype C from a cohort of 435 CHB patients without or with antiviral therapy. We found that 19 patients harbored the sC69^∗^ mutant. We then performed *in vitro* experiments to study the impact of the sC69^∗^ on viral replication, infectivity and spread and innate immune responses.

## Results

### Truncated Mutation at Site 69 in S Gene Tend to Occur in Mixed Population

Among 435 CHB patients, we identified 19 samples carrying HBV sC69^∗^ mutant by analyzing entire SHBs AA sequences. We grouped the patients into sC69 WT and sC69^∗^ groups (Table 1).

**Table 1:** Comparison of clinical data between patients with and without sC69^∗^ substitution.

Characteristics	Patients		*P*-value
	WT (416)	sC69^∗^ (19)	
Age (years), median (range)	36 (13–81)	46 (21–68)	0.004
Gender, male (%)	280 (67.3%)	13 (68.4%)	NS
LMV treatment (ratio%)	64 (15.4%)	2 (10.5%)	NS
ALT (U/L), median (range)	92.0 (24.1–1393.0)	87.0 (28.0–1150.0)	NS
AST (U/L), median (range)	70.0 (22.2–1207.0)	94.00 (39.0–985.0)	NS
HBV DNA (log_10_ IU/ml), median (range)	7.6 (3.1–10.6)	5.73 (3.9–9.4)	0.02
HBsAg (log_10_ IU/ml), median (range)	4.2 (2.4–5.5)	3.74 (3.0–4.2)	NS
HBeAg (positive%)	361 (86.8%)	14 (73.7%)	NS

*ALT, alanine aminotransferase; AST, aspartate transaminase; HBsAg, hepatitis B surface antigen; HBeAg, hepatitis B e antigen; NS, no significance; WT, wild-type.*

In order to know the distribution of sC69^∗^ mutant in different patient groups, we stratified patients by HBeAg status and LMV exposure and analyzed the SHBs sequences. This showed that the frequency of sC69^∗^ mutant varied in different CHB patients, including HBeAg-positive untreated patients (3.67%, 12/327), HBeAg-negative untreated patients (11.90%, 5/42) and LMV-treated patients (3.03%, 2/66) (Table 2). Of note, the sC69^∗^ significantly existed at a higher frequency in HBeAg-negative untreated CHB patients in comparison to that in HBeAg-positive ones (*p* < 0.05). In addition, we found that the sC69^∗^ mutant usually coexisted with WT sC69 shown by double peaks at the third nucleotide of the sC69 codon (TGT and TGA) in PCR direct sequencing electropherograms (Supplementary Figure S1A). Table 2 showed that 78.95% (15/19) of the sC69^∗^ mutations were identified as sC69C/^∗^ quasispecies, indicating that sC69^∗^ usually coexisted with the WT.

### The Negative Impact of the sC69^∗^ Mutant on Viral Infection Could Be Rescued by Co-expression of WT HBs

We showed before that the sC69^∗^ inhibits virion replication and secretion which can be rescued by co-expressing WT HBs to facilitate this mutant survival, indicating that WT surface proteins can package the sC69^∗^ mutant genome and allow the mutant to persist (Supplementary Figures S1B,C) (Xiang et al., 2017). The sC69^∗^ not only inhibits viral secretion, but also results in HBs locating around the hepatocyte nuclear (Supplementary Figure S1D).

**Table 2:** The prevalence of sC69^∗^ in HBV infection.

Patients	Number of patients	Codon at sC69		
		TGT (sC69) (%)	TGA (sC69^∗^) (%)	TGT/TGA (sC69C/^∗^) (%)
HBeAg-positive untreated	327	315 (96.33)	3 (0.92)	9 (2.75)
HBeAg-negative untreated	42	37 (88.10)	1 (2.38)	4 (9.52)
LMV-treated	66	64 (96.97)	0 (0.00)	2 (3.03)
Total	435	416 (95.63)	4 (0.92)	15 (3.45)

*HBeAg, hepatitis B e antigen; LMV, lamivudine.*

**FIGURE 1:**
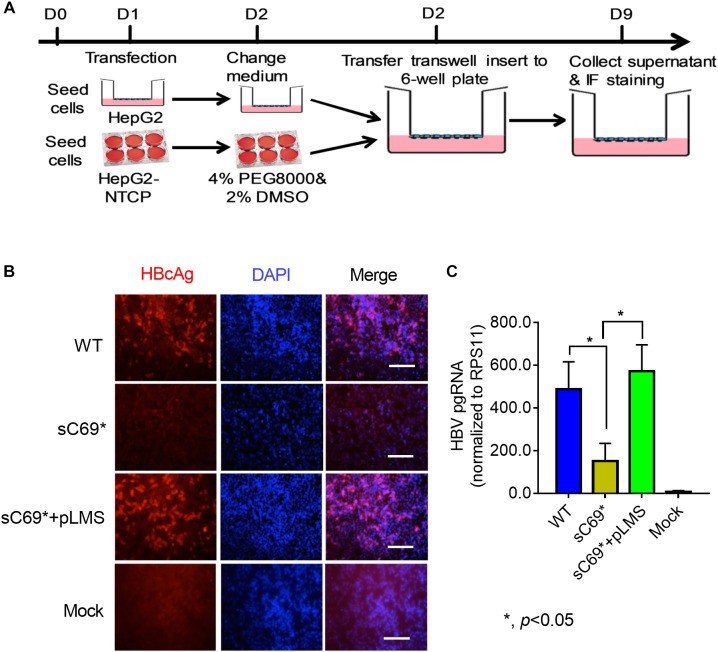
The viral infectivity affected by sC69^∗^ substitution could be rescued when co-existence with wild-type (WT) HBsAg. **(A)** The schematic strategy was used to test HBV infectivity in the *trans*-well system. To avoid the impact of collecting and concentrating steps on viral activity, we tried to use the *trans*-well system to test HBV infection. HepG2 cells seeded in the insert of *trans*-well were transfected with HBV WT, sC69^∗^ and sC69^∗^ + pLMS, respectively. The next day, the inserts were transferred to wells with HepG2-NTCP cells. Seven days post-infection, HBV related marker were detected. **(B)** Immunofluorescent microscopy of HBcAg in the WT, sC69^∗^ and sC69^∗^ + pLMS infection, scale bars: 100 μm. **(C)** RT-qPCR detection of HBV pgRNA at different groups. Data from three biological replicates are shown as means ± s.d. Statistical analysis was performed by a Student’s *t*-test ^∗^*p* < 0.05.

HepG2-NTCP cell line has been established (Supplementary Figure S2), which highly supported HBV infection and could be used to study sC69^∗^ *in vitro* in this study (Michailidis et al., 2017). To determine whether sC69^∗^ mutant could impact virion infection and spread, we modified the experimental system by using the transwell plate, in which the transfection-produced virions were generated in the upper insert and penetrated to the bottom well containing HepG2-NTCP cells (Figure 1A). Interestingly, WT HBV infection resulted in high positive infected cells, while the sC69^∗^ mutant showed no sign of infected cells. However, HepG2-NTCP cells infected by the virions produced by co-transfecting WT HBs expression plasmid pLMS and full viral genome encoding the sC69^∗^ mutant showed high percentage of hepatitis B core antigen (HBcAg) staining positive cells similar to that of the WT infection, indicating that WT HBs can package the sC69^∗^ mutant genome and help it infect new cells (Figure 1B). And also, *trans*-complementary experiment showed similar HBV pgRNA level to WT infection in HepG2-NTCP cells (Figure 1C). Taken together, the results suggested that the sC69^∗^ mutant could negatively impact viral infection and spread, while WT HBs could rescue sC69^∗^ mutant infectivity and spread. Thus, the sC69^∗^ + pLMS and WT were used in the following experiments.

### The Impact of the sC69^∗^ Mutant on Innate Immune Response in HepG2-NTCP Cell Line

**FIGURE 2:**
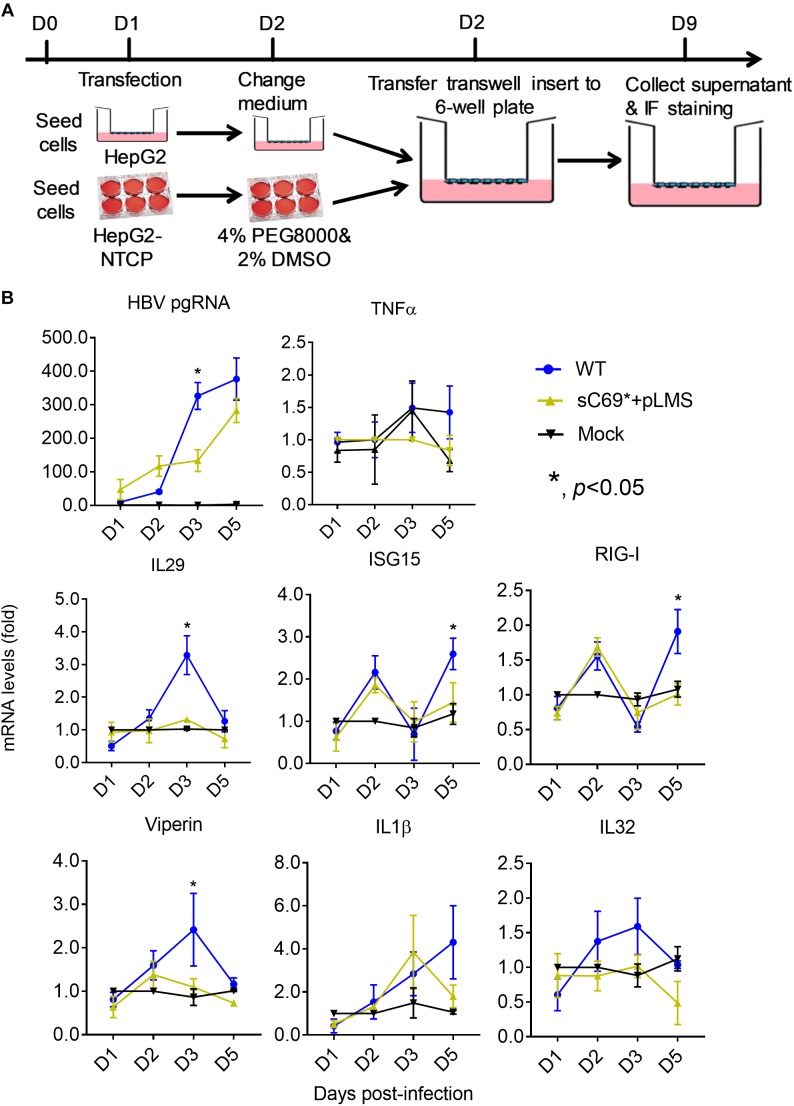
The sC69^∗^ mutant inhibits host innate immune response compared to HBV WT in HepG2-NTCP cells. **(A)** HepG2 cells seeded in the insert of *trans*-well were transfected with HBV WT and sC69^∗^ + pLMS, respectively. The next day, the inserts were transferred to wells with HepG2-NTCP cells. The cells were harvested at days 1, 2, 3, and 5 post-infection. **(B)** HBV pgRNA and innate immunity related mRNAs (TNFα, IL29, ISG15, RIG-I, viperin, IL1β, and IL32) were detected after the infection of HBV virions from transfection system. Data from three biological replicates are shown as means ± s.d. Statistical analysis was performed by a Student’s *t*-test ^∗^*p* < 0.05.

HepG2-NTCP cell line was competent for innate immune response with treatment of 10 μg/ml poly (I:C) overnight (Supplementary Figure S3), indicating that HepG2-NTCP cells could be stimulated to produce innate immune response. To ascertain whether sC69^∗^ could affect innate immune responses, we infected HepG2-NTCP cells in transwell system and quantified mRNA levels of innate immunity related genes (Figure 2A). As shown in Figure 2B, HBV pgRNA from WT and sC69^∗^ + pLMS increased over time. However, the pgRNA levels from WT are higher than sC69^∗^ + pLMS at day 3 of viral infection. Interestingly, many ISGs and cytokine mRNAs such as IL29, ISG15 and RIG-I (*p* < 0.05) were stimulated by WT HBV infection. However, when HepG2-NTCP cells were infected with sC69^∗^ + pLMS, most of ISGs and cytokines mRNAs were lower compared to WT at day 3 (IL29 and ISG15) and day 5 (RIG-I) (*p* < 0.05). In addition, their expression was not largely improved in sC69^∗^ + pLMS-derived virus infection, indicating that sC69^∗^ could inhibit innate immune responses to escape innate immune surveillance.

As shown before, the sC69^∗^ mutant could inhibit viral replication ability. To exclude the viral replication influence on the induction of innate immune response, we did RT mutation of HBV WT and sC69^∗^ from YMDD to YMHD (Supplementary Figure S4A). This mutation could inhibit viral replication. The expression of viral proteins and RNAs from the transfected plasmids would show similar levels for WT and sC69^∗^. Therefore, we could exclude the possibility that the less induction of innate immune response were caused by the less replication of sC69^∗^ mutant. We transfected WT-YMHD and sC69^∗^-YMHD into HepG2 cells and detect HBV pgRNA and ISGs expression at days 0.5, 1, 2, 3, 5, and 7 post-transfection. As shown in Supplementary Figure S4B, the expression of HBV pgRNA were almost the same between WT-YMHD and sC69^∗^-YMHD. However, some of the ISGs were still significantly higher 3 days post-transfection in WT-YMHD than those in sC69^∗^-YMHD, such as IL29, ISG15, RIG-I and viperin (*p* < 0.05). The expression of TNFα, IL1β, and IL32 was the same during the days post-transfection between WT-YMHD and sC69^∗^-YMHD. These results indicate that the less induction of innate immune response was not caused by less viral replication. Maybe the sC69^∗^ mutant itself antagonizes the innate immune response.

In order to study if WT and sC69^∗^ induce effective IFNs expression, we transferred the supernatant from WT and sC69^∗^ + pLMS infected HepG2-NTCP cells to new cells without infection and harvested the cellls 6, 12, 24, and 48 h post-transferring (Supplementary Figure S5A). We detected pgRNA and HBV total RNA levels from the infected cells. The data (Supplementary Figure S5B) showed that WT had higher pgRNA and total RNA expression than sC69^∗^ + pLMS, which meant that HepG2-NTCP cells supported well WT and lower sC69^∗^ mutant infection. However, when transferring the supernatants to new cells, we couldn’t find any ISGs expression, while the cells were induced with some ISGs expression under poly (I:C) treatment (Supplementary Figure S5C).

### The sC69^∗^ Mutant Attenuates the Innate Immune Response During Poly (I:C) Treatment

**FIGURE 3:**
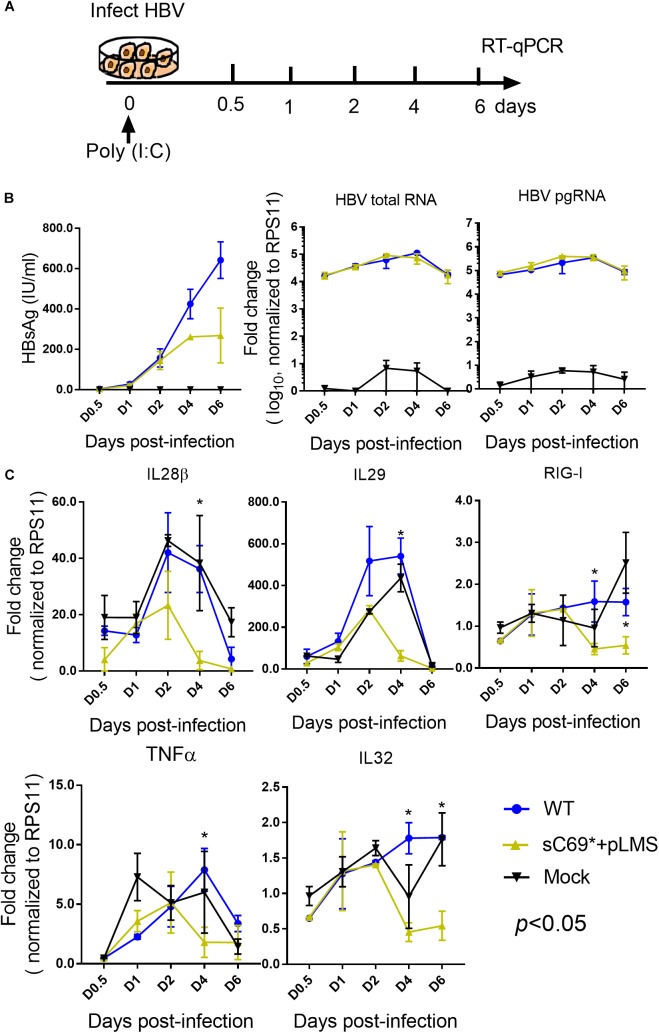
The sC69^∗^ mutant attenuates the poly (I:C) inducing host immune response in HepG2-NTCP cells. **(A)** Schematic workflow for detecting ISGs expression during HBV WT and sC69^∗^ + pLMS infection with poly (I:C) treatment in HepG2-NTCP cells. Briefly, we collected virions from HBV WT and sC69^∗^ + pLMS transfections that were used to, infect HepG2-NTCP cells with 10 μg/ml poly (I:C) during the infection and measured the viral and ISGs related marker 0.5, 1, 2, 4, and 6 days post-infection. **(B)** Representative expression of HBsAg, pgRNA, and HBV total RNA levels during the infection. **(C)** Expression of IL28β, IL29, RIG-I, TNFα, and IL32 mRNA levels were presented during the time course of viral infection. Data from three biological replicates are shown as means ± s.d. Statistical analysis was performed by a Student’s *t*-test ^∗^*p* < 0.05.

Given the very low induction of ISGs expression by sC69^∗^ + pLMS in HepG2-NTCP cells, we explored if sC69^∗^ mutant could attenuate poly (I:C) mediated immune response by monitoring the activation of ISGs expression. We stimulated HepG2-NTCP cells with 10 μg/ml poly (I:C) during HBV WT and sC69^∗^ + pLMS infection (Figure 3A). The levels of HBV pgRNA and total RNA of sC69^∗^ + pLMS was similar to those of WT (Figure 3B). Consistent to Figure 2, ISGs expression in the sC69^∗^ + pLMS infected cells was significantly lower compared to WT and poly (I:C) treated only cells (mock) (Figure 3C), suggesting that sCS69^∗^ could attenuate poly (I:C) stimulated ISGs expression.

**FIGURE 4:**
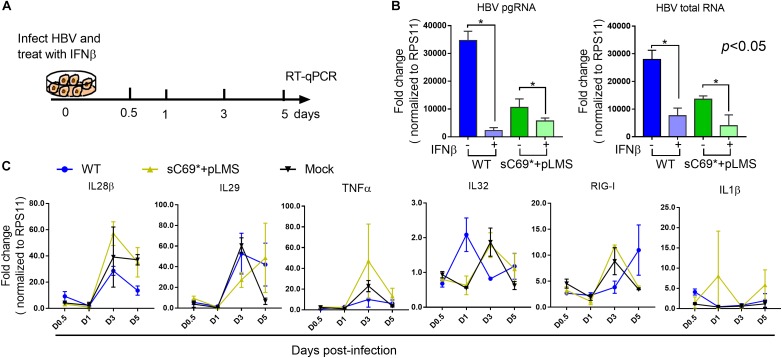
The sC69^∗^ has no impact on IFNβ induction of ISGs expression. **(A)** Schematic workflow for detecting identifying ISGs expression during HBV and sC69^∗^ + pLMS infection with IFNβ treatment. The HepG2-NTCP cells were infected by viruses of WT or sC69^∗^ + pLMS and treated with 500 U/ml of IFNβ during the infection. **(B)** HBV pgRNA and HBV total RNA were detected from cells 5 days post-infection. **(C)** Relative expression of innate immune response related genes in HepG2-NTCP cells with IFNβ treatment during the infection. Data from three biological replicates are shown as means ± s.d. Statistical analysis was performed by a Student’s *t*-test ^∗^*p* < 0.05.

To examine whether sC69^∗^ attenuates ISGs expression at early stage or late stage (IFNs mediated signaling) of ISGs induction, we stimulated WT and sC69^∗^ + pLMS infected HepG2-NTCP cells with 500 U/ml IFNβ during the infection and measured the expression of pgRNA, HBV total RNA and ISGs levels by RT-qPCR (Figure 4A). Consistent to previous study, expression of pgRNA and HBV total RNA in IFNβ untreated group were significantly higher than those with IFNβ treatment both in WT and sC69^∗^ + pLMS (Figure 4B). However, there were no difference of ISGs expression between WT and sC69^∗^ + pLMS (Figure 4C), indicating that sC69^∗^ attenuates of ISGs expression at early stage of innate immune response.

### *In vitro* HLCs Are Permissive for HBV Infection

To further confirm the novel findings obtained in HepG2-NTCP, we used HLCs derived from hESCs as a model to study HBV WT and mutant infection and related innate immune responses. Firstly, we investigated whether hESC-derived hepatocytes were permissive to HBV infection. The special markers for hESCs differentiation to HLCs were showed (Supplementary Figure S6). We used 500 geq/cell of HBV virions from HepDE19 cells to infect HLCs for 7 days. As shown in Supplementary Figure S7A, the HBV receptor of NTCP was highly expressed in these HLCs. HBcAg staining (Supplementary Figure S7B) and HBV pgRNA (Supplementary Figure S7C) from the cells, as well as HBsAg (Supplementary Figure S7D), HBeAg (Supplementary Figure S7E) and HBV DNA from the supernatant (Supplementary Figure S7F) were positive after HBV infection compared to negative control, indicating differentiated hepatocytes derived from hESCs were permissive for HBV infection.

### The Impact of the sC69^∗^ Mutant on Innate Immune Response in the HLCs Model

**FIGURE 5:**
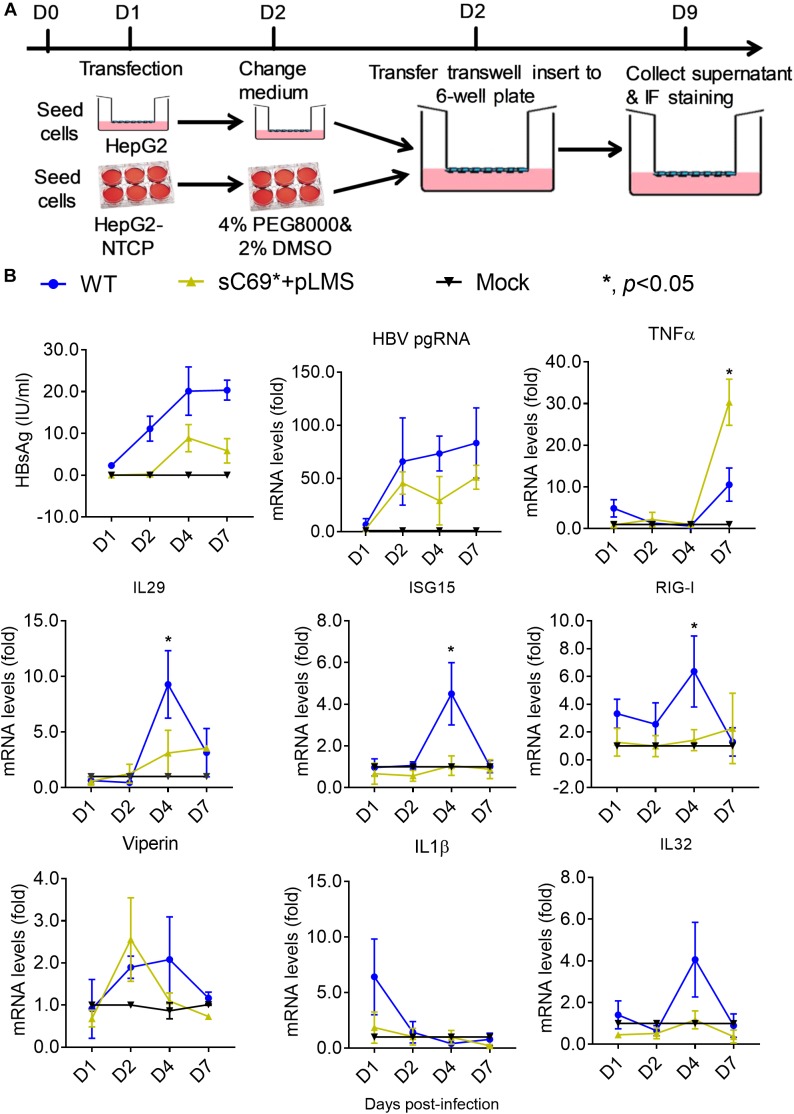
The sC69^∗^ mutant inhibits host innate immune response compared to HBV WT in *trans*-well system. **(A)** The schematic illustration of HBV WT and sC69^∗^ + pLMS infection. The HepG2 cells were seed in the insert of the transwell and the HLCs were prepared. We transfected HBV WT and sC69^∗^ + pLMS plasmid into HepG2 cells and washed the cells with PBS the next day. The day after, we transferred the insert to the top of HLCs and cultured as indicated. **(B)** CLIA detection for HBsAg and RT-qPCR for detecting HBV pgRNA and mRNAs of TNFα, IL29, ISG15, RIG-I, viperin, IL1β, and IL32 after 500 geq/cell HBV infection for different days. Data from three biological replicates are shown as means ± s.d. Statistical analysis was performed by a Student’s *t*-test ^∗^*p* < 0.05.

To study whether the sC69^∗^ mutant could affect innate immune responses; we carried out transwell-based infection experiment. Firstly, we transfected the WT and sC69^∗^ + pLMS into HepG2 cells seeded in the insert of the transwell. The next day we transferred the insert into plates seeded with HLCs and harvested the cells at different time points (Figure 5A). Interestingly, consistent with HBV infection in HepG2-NTCP system, the sC69^∗^ + pLMS showed significantly lower innate immune responses than WT regarding the expression of IL29, ISG15, and RIG-I mRNA levels 4 days post-infection and TNFα 7 days post infection (*p* < 0.05), while the other ISGs expression seemed to show overall lower trend in sC69^∗^ + pLMS than that of WT but without statistical difference (Figure 5B), indicating that sC69^∗^ could attenuate the expression of some ISGs.

**FIGURE 6:**
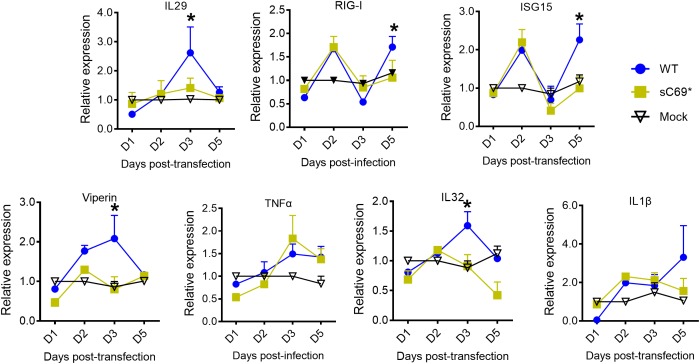
The truncated protein of sC69^∗^ attenuates the host innate immune response. RT-qPCR was used to detect the mRNA levels of IL29, ISG15, RIG-I, viperin, IL1β, and IL32. Data from three biological replicates are shown as means ± s.d. Statistical analysis was performed by a Student’s *t*-test ^∗^*p* < 0.05.

In order to study if the truncated protein attenuates the innate immune response, we used the plasmid of pLMS to study the impact of the truncated protein on innate immune response. We found that it has IFNs induction, with similar trend of ISGs expression to the replicon based results (Figure 6), indicating that the truncated protein could have an effect on the innate immune response.

## Discussion

In this study, we reported that the sC69^∗^ mutant was highly prevalent in the 435 CHB patients with and without antiviral treatment. Patients with the sC69^∗^ mutant were significantly older than those with WT infection. In addition, HBV DNA levels in patients with the sC69^∗^ mutant were significantly lower than those where sC69^∗^ was not detected. The sC69^∗^ mutant exists mostly in HBeAg-negative CHB patients and usually coexisted with WT as quasispecies. *In vitro* studies showed that the sC69^∗^ mutant could inhibit viral infection and spread, but could be rescued by WT surface protein. It was reported that HBsAg could be transcribed from HBV sequences that are integrated into the host genome rather than from replicating virus (Wooddell et al., 2017). In our study, we could detect HBV pgRNA levels, which supports the possible origin of sC69^∗^ from cccDNA. Therefore, our *in vitro* models should be able to resemble that occurring *in vivo* at least to some extent.

The sC69^∗^ mutant was reported in several published papers including our previous reports (Ding et al., 2014; Xiang et al., 2017). It not only exists in treatment-naïve patients, but also exists in LMV, ETV, and TDF treated patients (Xiang et al., 2017). However, in a recent study, Shirvani-Dastgerdi et al. (2017) reported that sC69^∗^ in genotype A HBV had a higher viral replication. The reason for this difference may be due to different aspects, such as genotype, the plasmid vector, HBV sequence, cell lines, etc. In our study, we found that both in patient serum and in *in vitro* studies the sC69^∗^ mutant showed lower HBV DNA levels than the WT HBV. In addition, there have been many other truncated HBsAg mutants reported by others showing similar results (Ding et al., 2014; Aragri et al., 2016). Lee et al. (2012) reported that sW182^∗^ mutant showed lower HBV DNA levels and existed preferentially in older patients.

There has been little investigation on the influence of HBs mutant on viral infectivity because available and efficient infection cell models are limited. In the past, analyzing how HBsAg mutants influenced HBV infection mostly relied on the hepatitis delta virus (HDV) model, in which HDV hijacks HBV surface proteins for assembly, release and entry into hepatocytes. It has been shown that sI100, sG119, sC124, sK141, sP142, sC147, and sC149 were critical for both virion formation and viral infectivity (Kwei et al., 2013). Recently, HepG2-NTCP and hESC-induced HLCs were reported to support HBV infection (Yan et al., 2015b; Seitz et al., 2016). We also established effective cell models for HBV infection and used them to study the impact of the sC69^∗^ mutant on viral infection and spread. In this study, we tested the impact of the sC69^∗^ on viral infectivity and spread in the HepG2-NTCP cell line. In addition, the *trans*-well system for HBV infection experiments was shown to be better than applying concentrated viruses directly onto susceptible cells for viral infection.

Interestingly, among 19 patients with the sC69^∗^ mutant, 15 patients mutation were found to have a mixture of WT sC69 and sC69^∗^ encoded by TGC + TGA. Thus, the questions came out why the sC69^∗^ coexisted with WT and how sC69^∗^ survived in the host. Based on our data, we proposed the following explanations. The WT HBV rescues the mutant and allows it to complete its life cycle. Warner and Locarnini (2008) showed that sW172^∗^ mutant could be rescued by WT for secretion. In addition, Kwei et al. (2013) reported that mutants in the ‘a’ determinant were also rescued by WT to secrete out of the cells. As shown in our previous report, the sC69^∗^ mutant had impaired replication and secretion (Xiang et al., 2017). In this study, we also found that sC69^∗^ alone also resulted in deficient virion infection and spread in HepG2-NTCP. The potential mechanism may be that the mutant had truncated HBsAg and negatively affected virion secretion. More studies are needed for further understanding.

In addition, HBV infection in HepG2-NTCP and HLCs models showed an innate immune response during the infection, indicating that these two models can be used to study innate immune responses during HBV infection. However, sC69^∗^ could inhibit some immune factors expression (RIG-I, IL29, and ISG15) with or without WT. Moreover, the sC69^∗^ mutant could attenuate host innate immune responses to help the WT and itself to escape the host immune surveillance. Thus, the WT and mutant can survive through cooperation relationship in the host. Liu S. et al. (2015) reported that HBsAg could inhibit major vault protein signaling in IFN induction pathways. It is still not clear if HBsAg mutants impact the host immune response or not. We suspect that the sC69^∗^ mutant might show stronger inhibition on the innate immune response than WT. In this study, we used both the HepG2-NTCP cell line and HLCs derived from hESCs as HBV infection systems to study the sC69^∗^ mutant influence on innate immune responses. The results showed that sC69^∗^ might have stronger inhibition on the innate immune responses compared to that of the WT HBV. As we know, this should be the first report to relate HBsAg mutant to innate immune responses. When infected HepG2-NTCP cells were treated with poly (I:C), the pgRNA and HBV total RNA levels of sC69^∗^ were similar to WT. These indicate that sC69^∗^ mutant could persist in the host through escape host innate immune response. They could also use the cooperation strategy to work together to get better viral fitness and persistence. The less innate immune response of sC69^∗^ mutant was shown not to be caused by its lower replication, which lead to less virus production. It is more likely that the lower innate immune response is as a result of the surface protein being truncated. However, the issue of how sC69^∗^ inhibits host innate immune response remains to be elucidated in a future study.

Other publications reported that HBV did not induce any innate immune response (Cheng et al., 2017; Mutz et al., 2018; Suslov et al., 2018). Our data suggest that WT HBV could slightly induce innate immune response, although we could not detect ISG-related proteins by Western Blot. Of note, the virus we used is genotype C rather than genotype D or A virus that was used in other studies, which may lead to different IFN responses. Our results are similar to those in other studies (Sato et al., 2015). The sC69^∗^, with the concomitant rtS78T mutation impaired viral replication *in vitro*, which may explain the association of sC69^∗^ with low HBV DNA levels in patient sera. Moreover sC69^∗^ was found to inhibit expression of a number of ISGs *in vitro* and this may possibly lead to attenuation of the innate immune response.

There are some limitations in this study. As mentioned above, some of our results were different from other studies, such as the influence of sC69^∗^ on viral replication (Shirvani-Dastgerdi et al., 2017). There are perhaps many reasons for this difference, for example the different HBV sequence, plasmid vector, cell lines for HBV replication and infection, genotypes and detection methods (Shirvani-Dastgerdi et al., 2017). In addition, the infection efficiency in HLCs was also low with about 40% of infected cells, which maybe impact HBV infection and innate immune responses. In addition, how sC69^∗^ mutant antagonizes immune response is needed to be studied in the future.

## Conclusion

We found that sC69^∗^ usually coexists with WT HBV. The sC69^∗^ substitution could impact viral infection and be rescued when coexisting with WT. In addition, sC69^∗^ could possibly inhibit the innate immune response compared to the WT, allowing the virus to escape immune surveillance.

## Materials and Methods

### Serum Samples

Serum samples from 435 CHB patients with genotype C HBV infection were used, which were stored at −80°C from our previous studies (Hao et al., 2015; Liang X. et al., 2015; Su et al., 2016). 369 samples were from treatment-naïve patients (327 HBeAg-positive and 42 HBeAg-negative). 66 samples were from patients with ongoing lamivudine (LMV) treatment. The written informed consents were obtained from the participants of this study. All patients were free for hepatitis C virus, hepatitis D virus, human immunodeficiency virus, auto-immune liver disease and alcohol/drug abuse. The serological markers for HBV infection were detected as mentioned in our previous study (Xiang et al., 2017).

### PCR Amplification, DNA Sequencing and Sequence Analysis

As described before, we amplified HBV RT sequences covering the entire S gene and sequenced to identify the AA substitutions in SHBs (Ding et al., 2014).

### Plasmids

We cloned the sC69^∗^ mutant into plasmid pBB4.5 1.2/PC, which contained a 1.2-fold length HBV genome of genotype C with a G1896A mutation in the precore region (Xiang et al., 2017). HBV replication deficient mutation of YMDD to YMHD for WT-YMHD and sC69^∗^-YMHD were cloned based on the WT and sC69^∗^ mutant (Xiang et al., 2017). Hemagglutinin (HA) tag was inserted into the C terminus of surface protein of the WT and after AA68 of sC69^∗^ mutant surface protein as described before (Xiang et al., 2017). As shown before, a pBluescript II KS (+) plasmid (Addgene, United States) was used to express HBsAg. In brief, the LHBs coding region was inserted into the *KpnI* and *SacI* sites of this vector, which supported expression of SHBs, MHBs, and LHBs and was named as pLMS (Xiang et al., 2017).

### HBV Inoculum Preparation

Infectious HBV particles were collected from HepDE19 cells (a gift from Haitao Guo, Indiana University) as previously described (Michailidis et al., 2017). Briefly, HepDE19 cells were cultured in tetracycline-free medium to induce HBV virion production. Supernatant was collected every other day and replenished for 20 days. The collected supernatant was concentrated with Centricon^®^ Plus-70 Filter (Millipore, United States) by centrifuging with 2,500 × *g* for 30 min at 4°C. The concentrated HBV particle stocks were stored at −80°C. HBV inoculum was determined by quantitative PCR (qPCR) of HBV DNA. The quantification of HBV inoculum was expressed as HBV genome equivalent/cell (geq/cell), which was equal to HBV DNA copies. The protocol for HBV DNA quantitation was described before (Shlomai et al., 2014; Xiang et al., 2017).

### Differentiation of Human Embryonic Stem Cells (hESCs) Into Induced Hepatic-Like Cells (HLCs)

The protocol for hESCs (H1) differentiation into HLCs was described before (Wu et al., 2018). Confluent hESCs were dissociated into single cells with accutase and seeded into 12-well plates cultured with mTeSR^TM^ (knock out DMEM/F-12 with 5% hESC supplement, StemCell Technologies, Vancouver, Canada). Definitive endoderm (DE) was induced with the Definitive Endoderm Kit (StemCell Technologies, Vancouver, Canada) following the manufacturer’s instructions. To differentiate cells to hepatic progenitors, the cells were cultured in the presence of RPMI/B-27 supplemented with 20 ng/ml BMP4 and 10 ng/ml bFGF for 5 days. Then, cells were exposed to RPMI/B-27 with 20 ng/HGF for 5 days. The immature hepatocyte-like cells were cultured in the presence of HCM (Lonza, Switzerland) with 20 ng/ml oncostatin M for 5 days with changes of medium every 2 days.

### HBV Infection

HepG2-NTCP and HLCs cells were used for HBV infection. The infection protocol was shown before (Michailidis et al., 2017). The poly (I:C) (Sigma, Shanghai, China), and IFNβ (PeproTech, Suzhou, China) were used to treat the cells during HBV infection. The details were showed at each experiment.

### HBV Infection in *Trans*-Well System

Both HepG2-NTCP and HLCs were used for studying the sC69^∗^ mutant with WT as a control *in vitro*. HepG2 cells were seeded to the top and HepG2-NTCP or HLCs were seeded in the bottom of the 24-well *trans*-well plates, respectively. The HepG2-NTCP cells were treated with 2% DMSO 1 day before infection. As described before, plasmids were transfected into HepG2 cells in the presence of X-tremeGENE 9 at a ratio of plasmids and transfection reagent 0.5 μg:1.5 μl (Roche Diagnostics, Mannheim, Germany) (Xiang et al., 2017). 12 h later, the cells were washed five times with phosphate buffered saline (PBS) and then kept in fresh medium. Then, the inserts were transferred into HepG2-NTCP or *HLCs* *trans*-well plates and the bottom medium were replaced with fresh medium containing 4% PEG8000 and cultured for 5 or 7 days (Yan et al., 2015b).

### Immunofluorescence Assay

Cells were fixed with 4% paraformaldehyde diluted in PBS for 20 min and permeabilized with 0.1% Triton-X 100 diluted in PBS for 10 min at room temperature. Then cells were blocked in 10% goat serum with 1% bovine serum albumin (BSA) for 60 min on the shaker, followed by anti-HBc (Austral, United States), anti-HA (Sigma, United States), anti-nanog (Santa Cruz, Dallas, TX, United States), anti-FoxA2 (Sigma-Aldrich, St. Louis, MO, United States), anti-HNF4a (Cell Signaling), anti-AFP (Sigma-Aldrich, St. Louis, MO, United States) or anti-ALB (Cedarlane, Burlington, Canada) antibodies in 1:500 dilution with 1% BSA in PBS incubated at 4°C over night. The next day, the primary antibody was washed away and the 594 or 488 fluorescent anti-rabbit secondary antibody and 5 mg/ml 4′,6-DAPI with 1:20,000 dilution were added and incubated at 37°C for 30 min. Then, after washing the secondary antibody away, the cells were observed by fluorescent microscopy (Wu X. et al., 2012).

### Quantification Analysis of HBV pgRNA and Host Genes

The quantitative reverse transcription PCR (RT-qPCR) for HBV pregenomic RNA (pgRNA) and host gene expressions were performed as previously described (Shlomai et al., 2014; Xiang et al., 2017). In brief, total HBV RNA was isolated and quantified. First strand cDNA was synthesized using SuperScript III RT kit (Invitrogen). Ribosomal protein S11 (RPS11) gene was used to normalize RT-qPCR results for HBV transcripts. The expression of the host genes tested in this study included IL29, ISG15, RIG-I, IL-1b, IL32, viperin, TNF-α, etc., which reflected the host innate immune responses to HBV infection. The primers were shown in Supplementary Table S1.

### Detection of HBsAg and HBeAg

The HBsAg and HBeAg detection were as previously described (Xiang et al., 2017). In brief, serum HBsAg levels were detected by the Architect HBsAg QT (Abbott) Assay. HBsAg and HBeAg levels from cell culture supernatants were detected with commercial CLIA kits (Autobio Diagnostics Co., China).

### Statistical Analyses

Statistical analysis was performed using the SAS version 9.1 software package. Continuous and categorical variables were compared between groups using the student’s *t*-test and Chi-square test. Values of *p* < 0.05 were considered statistically significant.

## Ethics Statement

Both the informed and written consents were obtained from the participants of this study. One participant was under the age of 16, whose written consent was obtained from his parent.

## Author Contributions

TL and HZ created the study concept and design. KX, YX, YL, LH, and LW acquired the data (sample collection, processing, database establishment, etc.). KX and TL analyzed and interpreted the data. KX provided statistical analysis and drafted the manuscript. TL and HZ critically revised the manuscript for important intellectual content.

## Conflict of Interest Statement

The authors declare that the research was conducted in the absence of any commercial or financial relationships that could be construed as a potential conflict of interest.

## Abbreviations

AA: amino acid
AFP: alpha-fetoprotein
Alb: albumin
ALT: alanine aminotransferase
AST: aspartate aminotransferase
cccDNA: covalently closed circular DNA
CHB: chronic hepatitis B
CLIA: chemiluminescence assay
DAPI: 4′,6-diamidino-2-phenylindole
DMSO: dimethyl sulfoxide
HBsAg: hepatitis B surface antigen
HBV: hepatitis B virus
HCM: hepatocyte culture medium
hESCs: human embryonic stem cells
HGF: hepatic growth factor
HLCs: induced pluripotent stem cell derived hepatocyte-like cells
ISGs: interferon stimulated genes
IU/ml: international unit per milliliter
LHBs: large hepatitis B surface protein
LMV: lamivudine
MHBs: middle hepatitis B surface protein
MHR: major hydrophilic region
NTCP: sodium taurocholate cotransporting polypeptide
qHBsAg: quantitative hepatitis B surface antigen
RT: reverse transcriptase
SHBs: small hepatitis B surface protein
WT: wild-type.
