# Supplementary figures and images for: The Effect of the Hepatitis B Virus Surface Protein Truncated sC69∗ Mutation on Viral Infectivity and the Host Innate Immune Response
Source: Front Microbiol. 2019 Jun 12;10:1341. doi: 10.3389/fmicb.2019.01341 (PMC6584109; doi:10.3389/fmicb.2019.01341)

Figure S1

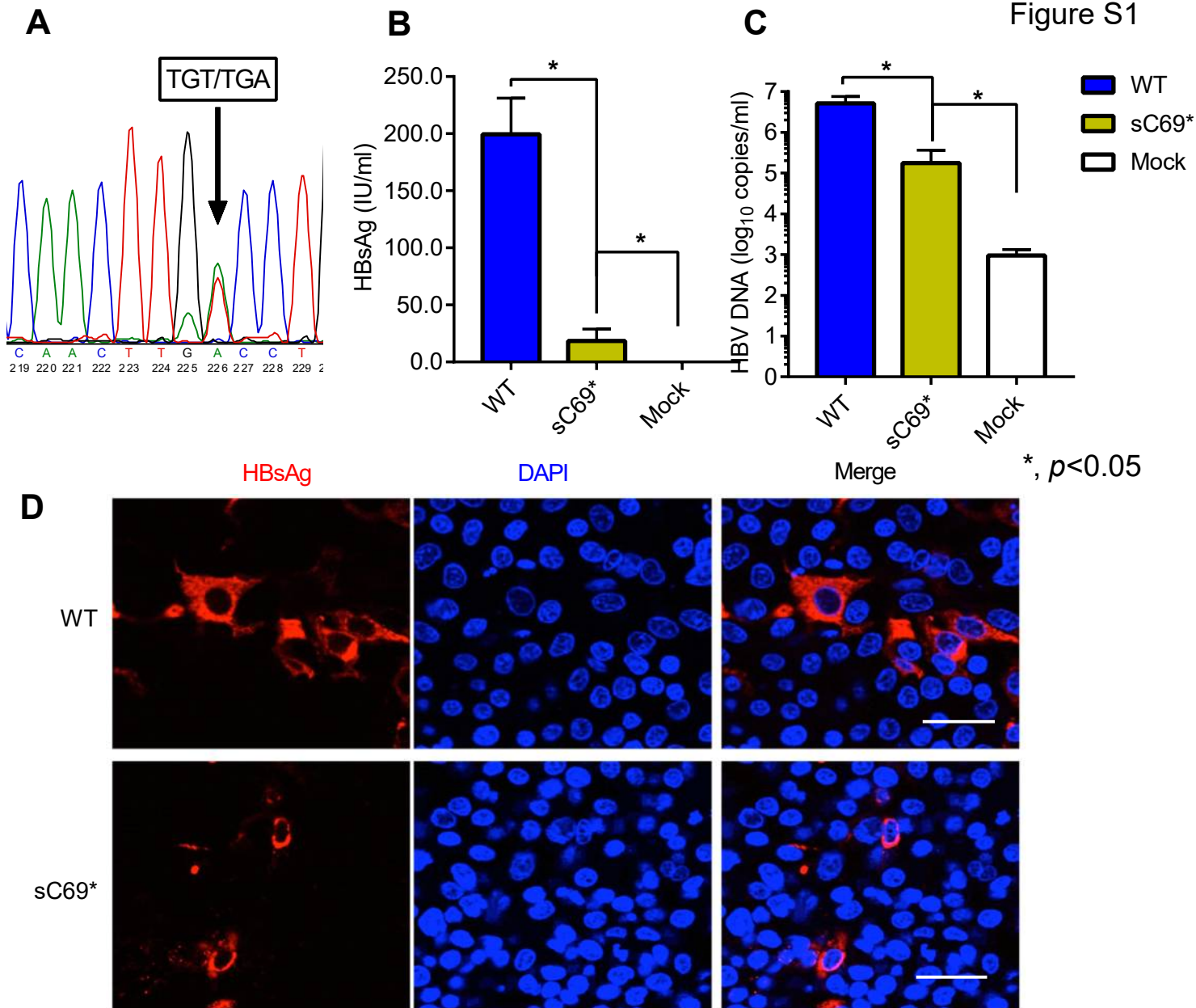

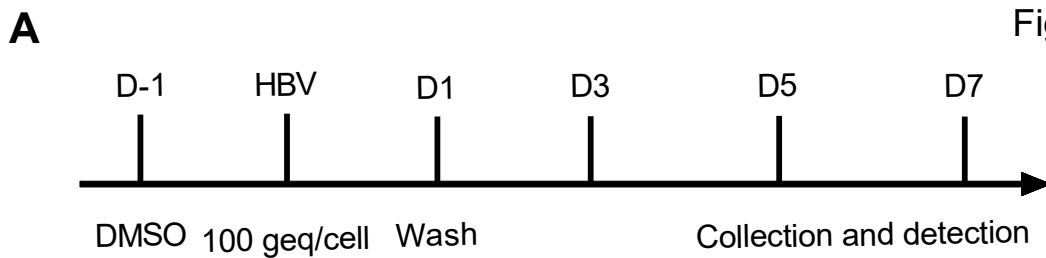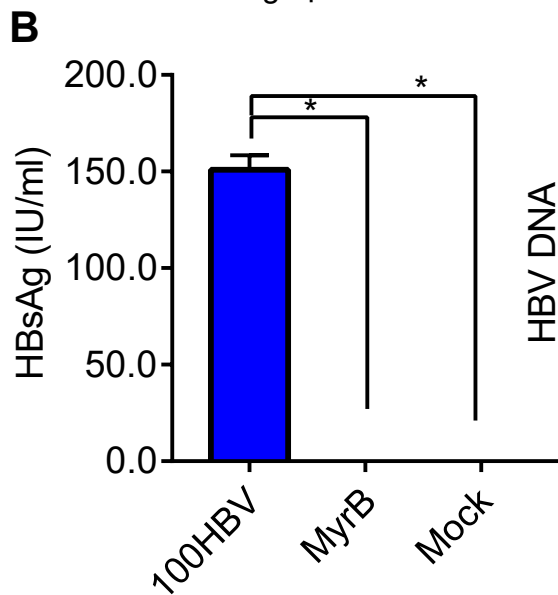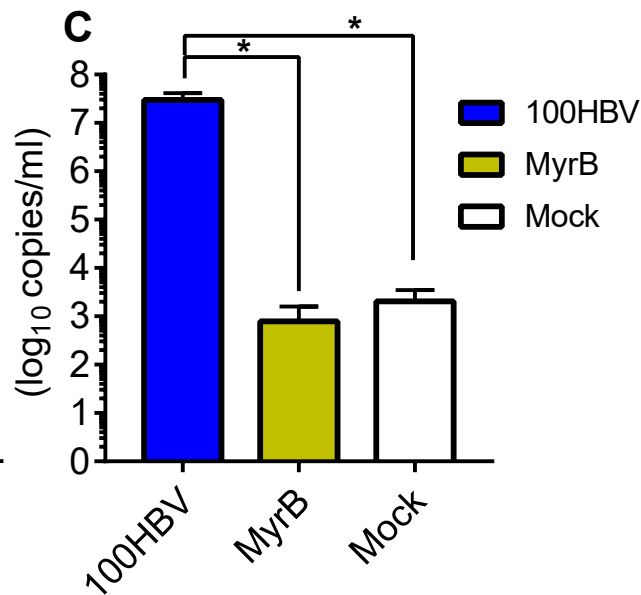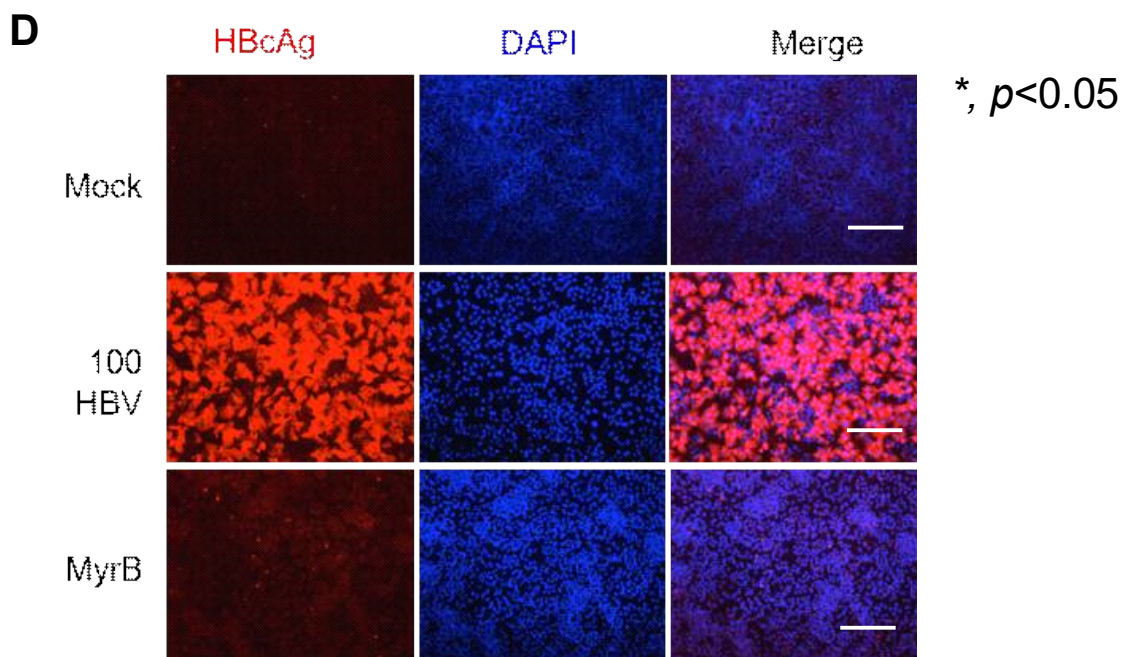

Figure S3

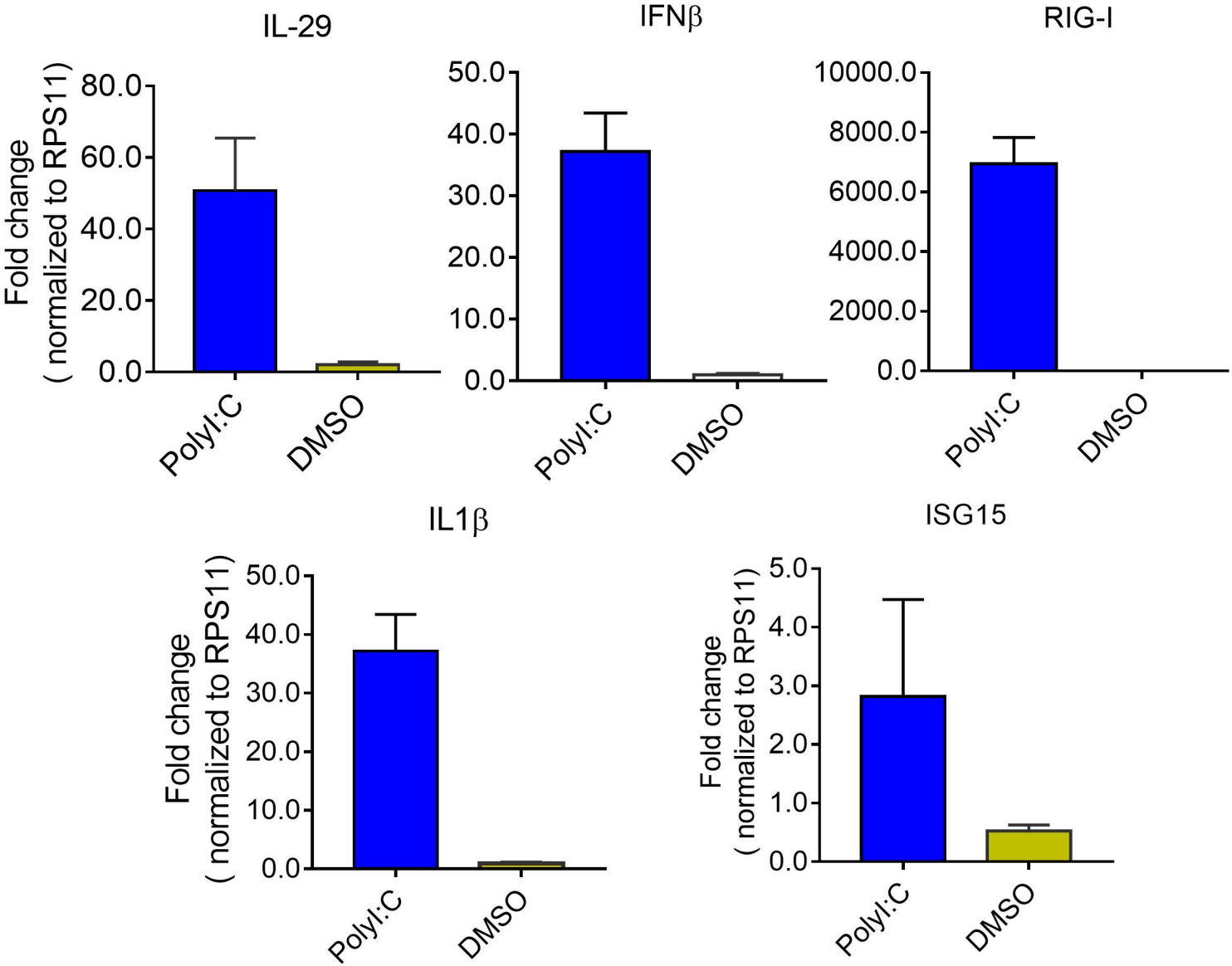

**A** Figure S4

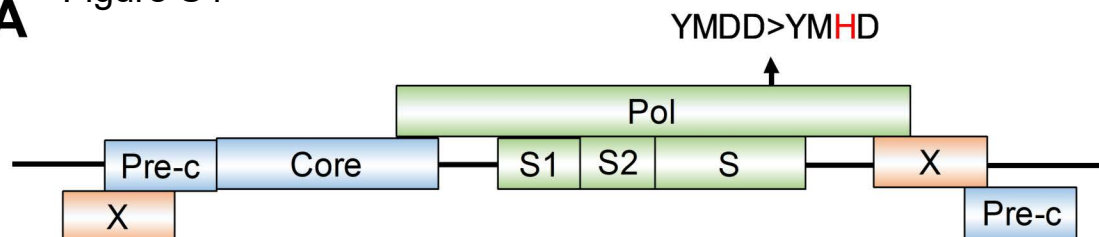

**B**

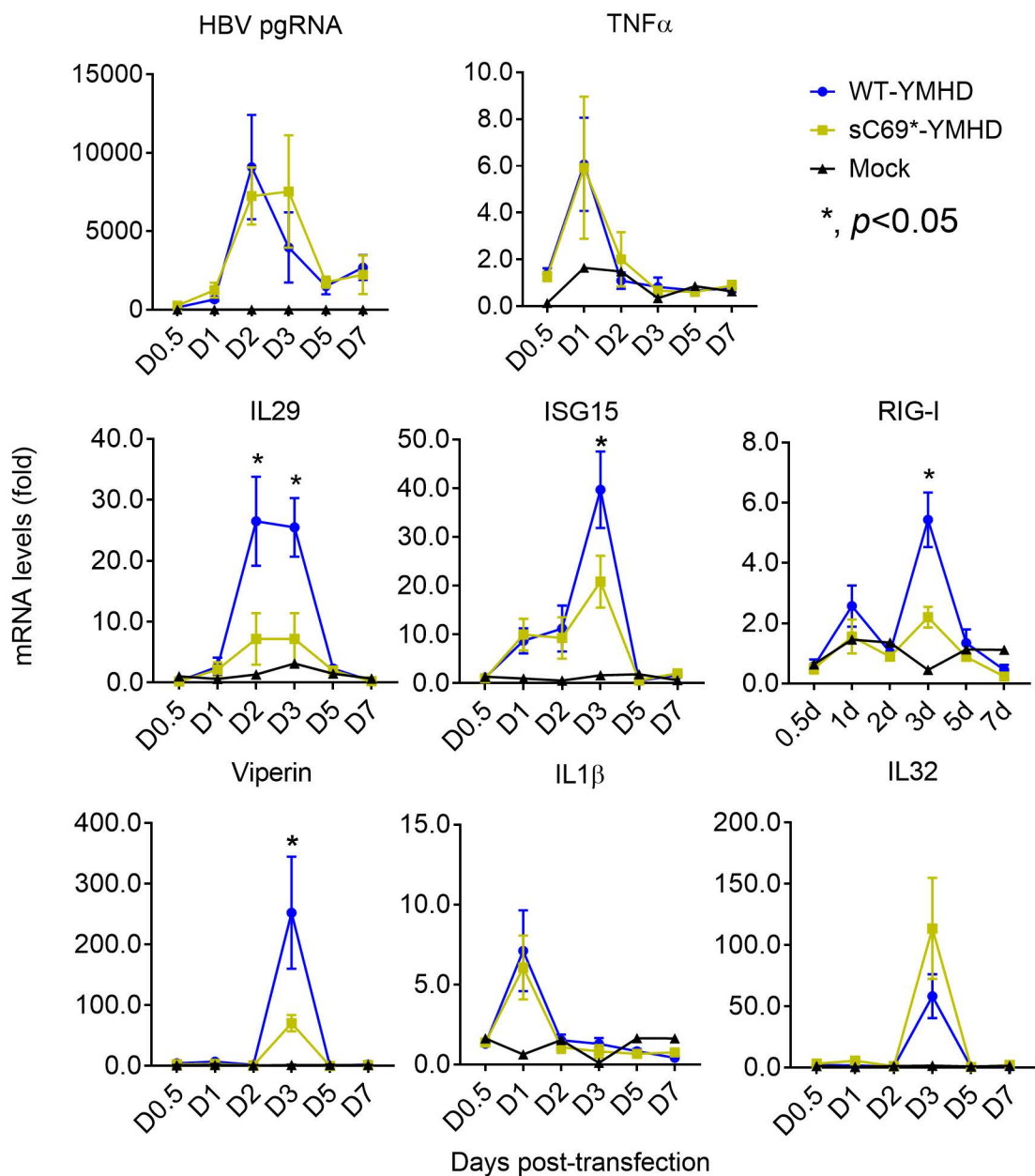

**A** Figure S5

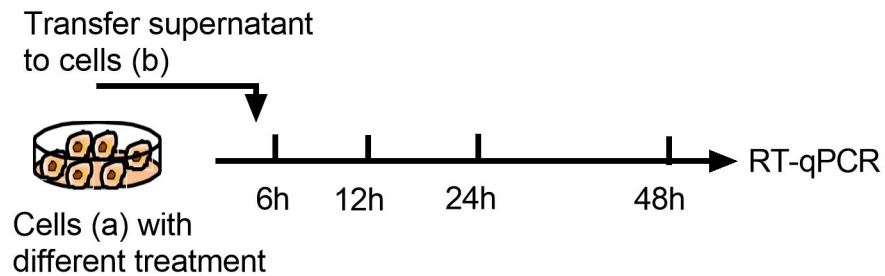

**B**

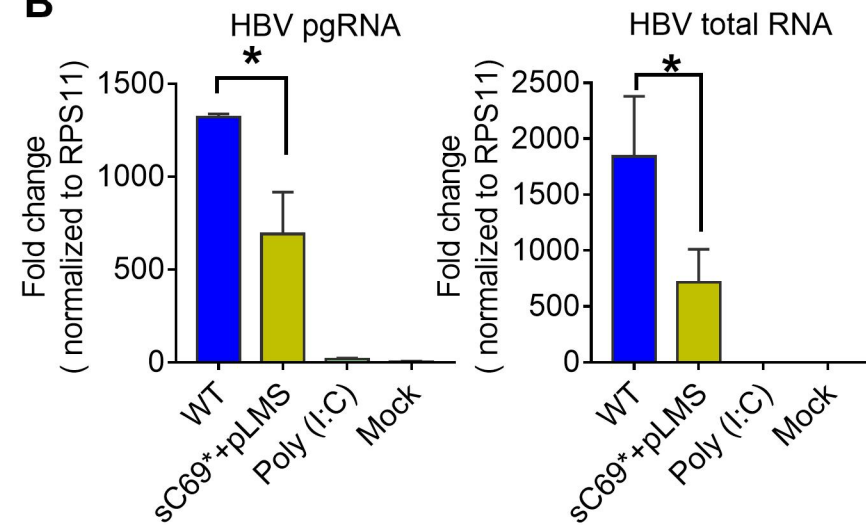

**C**

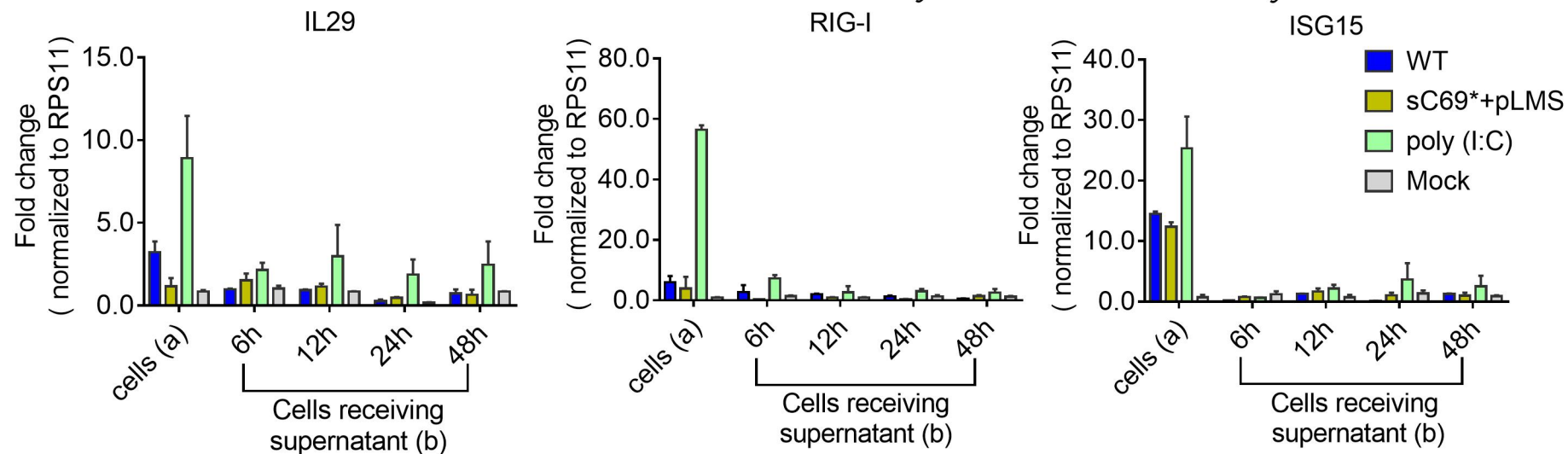

Figure S6

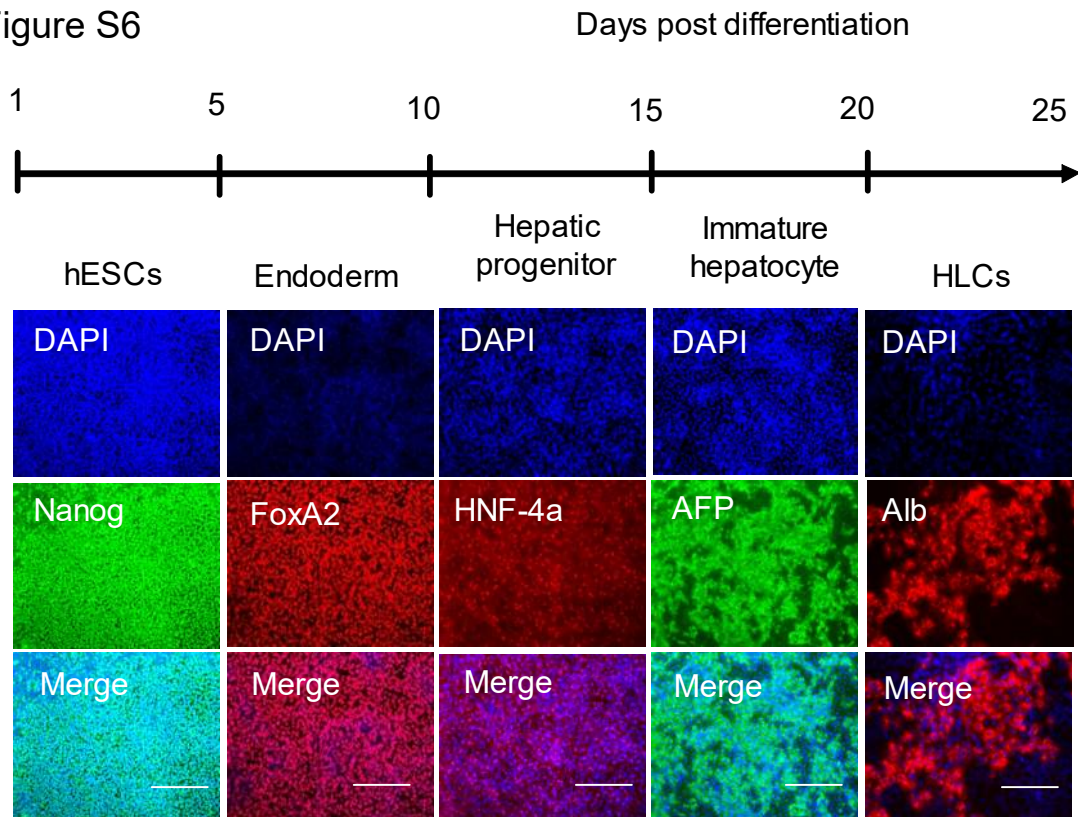

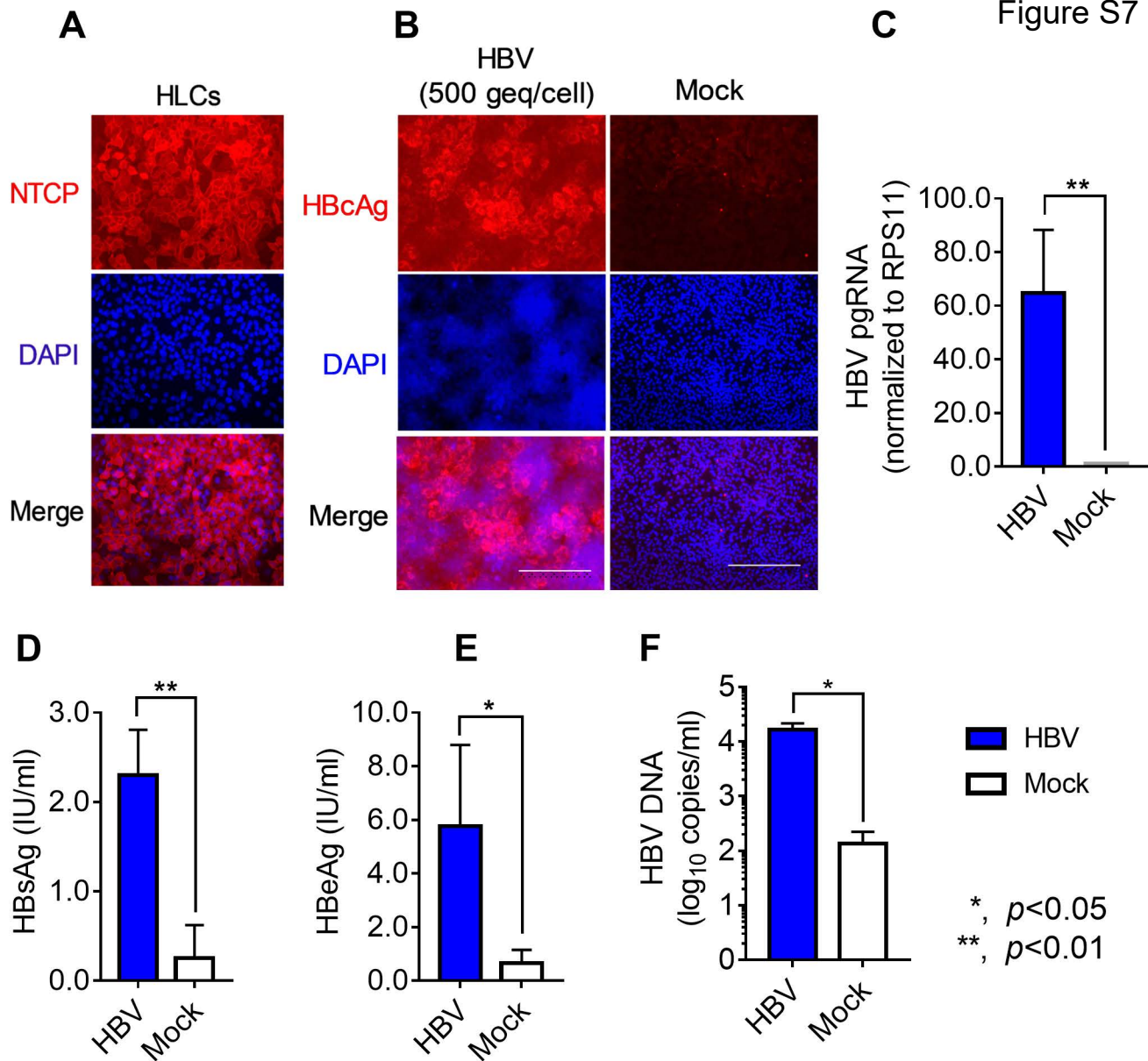

Supplement: Supplementary file 1 [file Data_Sheet_1.pdf]
