# Supplementary material for: The Effect of the Hepatitis B Virus Surface Protein Truncated sC69∗ Mutation on Viral Infectivity and the Host Innate Immune Response
Source: Front Microbiol. 2019 Jun 12;10:1341. doi: 10.3389/fmicb.2019.01341 (PMC6584109; doi:10.3389/fmicb.2019.01341)
Supplement: Supplementary file 2 [file Table_1.docx]

**Supplementary figure legends**

**Fig.S1. The localization of S protein from sC69* mutan**t. To visualize the cellular localization of the truncated S protein of sC69*, we transfected the WT and sC69* with HA tag into the HepG2 cells. 72 hours later, HBV related markers were detected. (A) The co-existence of sC69* substitution (TGA) and WT (TGT) in HBV SHBs sequence from a patient’s serum. (B) CLIA detection of the extracellular HBsAg. (C) qPCR detection of extracellular HBV DNA. (D) Immunofluorescence microscopy observation to detect the location of HBsAg-HA, scale bars: 100 μm. Data from three biological replicates are shown as means ± s.d. Statistical analysis was performed by a Student’s t test *: *p* < 0.05.

**Fig. S2. The establishment of HepG2-NTCP cells for HBV infection.** (A) Schematic workflow of HBV infection in HepG2-NTCP cells. Briefly, one day before 100 geq/cell of HBV infection, the cells were treated with 2% DMSO. The entry inhibitor MyrB was used to treat the cells at concentration of 500 nM. The next day, the cells were washed with PBS for 5 times to wash the left viruses away. Then, HBV related markers were measured after 7 days infection. Representative expression of (B) HBsAg and (C) HBV DNA levels were tested by CLIA and qPCR. (D) Representative image of HepG2-NTCP cells seven days after infection. Cells were stained with anti-HBc antibody and analyzed by immunofluorescence assay. Data from three biological replicates are shown as means ± s.d. Statistical analysis was performed by a Student’s t test *: *p* < 0.05.

**Fig. S3. HepG2-NTCP cells were stimulated by poly (I:C) with high ISGs expression**. HepG2-NTCP cells were treated with 10 μg/ml poly (I:C) for 24h. The ISGs related markers were measured by RT-qPCR. Data from three biological replicates are shown as means ± s.d.

**Fig.S4. The less immune response of sC69* is not caused by the deficient replication.** (A) The schematic workflow of HBV WT and sC69* reverse transcriptase mutation from YMDD to YMHD. (B) The plasmids of WT-YMHD and sC69*-YMHD were transfected into HepG2 cells. The expression of HBV pgRNA and TNFα, IL29, ISG15, RIG-I, viperin, IL1β and IL32 were detected by RT-qPCR at different days post-transfection. Data from three biological replicates are shown as means ± s.d. Statistical analysis was performed by a Student’s t test *: *p* < 0.05.

**Fig. S5. Poly (I:C) induce cytokines triggering ISGs expression in specific HepG2-NTCP cells**. (A) Schematic strategy for testing HBV WT and sC69* inducing ISGs expression. Supernatant from HBV WT and sC69*+pLMS infected cells were transferred to new HepG2-NTCP cells. Poly (I:C) was used as positive control for this experiment. The markers relating to innate immune response were measured by RT-qPCR at 6, 12, 24 and 48 hours post transfer. (B) HBV pgRNA and HBV total RNA expression were measured by RT-qPCR. (C) Expression of ISGs was measured by RT-qPCR. Data from three biological replicates are shown as means ± s.d. Statistical analysis was performed by a Student’s t test *: *p* < 0.05.

**Fig. S6. Schematic workflow for hESCs differentiation to HLCs**. The hESCs cells were differentiated to endoderm with special media for five days, and then another five days were used for differentiation to hepatic progenitor. After that, hepatic related factors were used for differentiating to immature hepatocyte for five days then another five days to HLCs. The cells at different stages of differentiation were stained with indicated antibodies and analyzed by immunofluorescence assay. scale bars: 100 μm.

**Fig. S7. Differentiated hepatocytes derived from human embryonic stems cells (hESCs) are permissive for HBV infection.** The induced hepatocytes were infected with 500 geq/cell HBV viruses for seven days, then immunofluorescent microscopy observation for detecting (A) NTCP and (B) HBcAg, scale bars: 100 μm and (C) RT-qPCR for detecting HBV pgRNA seven days post-infection. CLIA detection of (D) HBsAg and (E) HBeAg. (F) HBV DNA levels of HBV infection in differentiated hepatocytes. Data from three biological replicates are shown as means ± s.d. Statistical analysis was performed by a Student’s t test *: *p* < 0.05. **, *p* < 0.01.

Supplementary table 1. Primers used for RT-qPCR

| Gene | Forward primer (5’---3’) | Reverse primer (5’---3’) |
| --- | --- | --- |
| pgRNA | GAGTGTGGATTCGCACTCC | GAGGCGAGGGAGTTCTTCT |
| HBV total RNA | AAGCCACCCAAGGCACAG | GCACCAGCACCATGCAAC |
| RPS11  IL28β | GCCGAGACTATCTGCACTAC  TAAGAGGGCCAAAGATGCCTT | ATGTCCAGCCTCAGAACTTC  CTGGTCCAAGACATCCCCC |
| IL29 | GTGACTTTGGTGCTAGGCTTG | GCCTCAGGTCCCAATTCCC |
| TNFα | ATGAGCACTGAAAGCATGATCC | GAGGGCTGATTAGAGAGAGGTC |
| RIG-I | GATGCCCTAGACCATGCAGG | GCCATCATCCCCTTAGTAGAGC |
| IL32 | TGAGGAGCAGCACCCAGAGC | CCGTAGGACTGGAAAGAGGA |
| Viperin (RSAD) | TTCACTCGCCAGTGCAACTAC | CGGTCTTGAAGAAATGGTCT |
| IL1β | TGTAATGAAAGACGGCACACC | TCTTCTTTGGGTATTGCTTGG |
| ISG15 | GCGCAGATCACCCAGAAGAT | GTTCGTCGCATTTGTCCACC |
